# Book Review: New Perspectives on Willingness to Communicate in a Second Language

**DOI:** 10.3389/fpsyg.2021.715684

**Published:** 2021-06-25

**Authors:** Chao Lei

**Affiliations:** School of Foreign Languages, Zhoukou Normal University, Zhoukou, China

**Keywords:** book review, second language, education, WTC, cultural differences

Willingness to communicate (WTC) in second (L2) or foreign language education has received considerable attention within the field of second language acquisition and applied linguistics. Some scholars have scrutinized the conceptual and empirical cornerstones of the dynamic nature of the WTC model posited by MacIntyre et al. (1998). It is stipulated that WTC has an undeniable impact on the learners' level of L2 motivation, success, and engagement; therefore, *New Perspectives on Willingness to Communicate in a Second Language*, edited adeptly by Nourollah Zarrinabadi and Mirosław Pawlak, is an opportune volume that successfully and innovatively looks at WTC in L2 from diverse theoretical perspectives and rigorous methodological studies to unearth the antecedents and predictors, facilitating, or debilitating learners' WTC in L2.

This collection encompasses 13 chapters. In the introductory chapter, the editors succinctly justify the role of WTC in L2 and outline the structure of the book. Embarking on the complex dynamic system, Chapter 2 conceptualizes WTC and argues that WTC is a multifaceted concept that needs to be scrutinized from different perspectives. Chapter 3, drawing on the developmental and qualitative study investigating the role of WTC among Iranian migrants in their past English classes and their new experiences in their present classes in New Zealand, concludes that such factors as “family influence, type of school, and teacher expertise” (p. 25) affected their WTC in the Iranian context while in New Zealand “their relationships with their classmates, opportunities to speak in and out of class, and the effect of different types of curriculum” (p. 25) affect their WTC.

The focus of Chapter 4 is to report the German EFL expatriates' WTC in how they experience their sojourn abroad, and how cultural differences can affect their intercultural communication, which affect their foreign language learning. The findings substantiate that utilizing English as a lingua franca has a decisive role in expatriates' communication and boosts their WTC. One of the merits of this chapter is the triangulation of the data, shedding more light on the ecologically-dependent and dynamic nature of WTC. One of the genuinely engaging chapters is Chapter 5 that aims to explore how cultural differences can mediate between self-assessment of language skills and WTC among Italian and Polish learners. The chapter elucidates that “the Italian participants not only assessed their level of English subskills significantly higher than the Polish students, but also that they were more willing to communicate in both settings” (p. 85). Although investigating the role of cross-cultural factors is a worthwhile contribution, if the author had collected qualitative data, the findings would have enlightened the readers more.

Chapter 6 aims to unravel what learners' WTC and reticence signify to their English language teachers and explore the antecedents and causes of WTC and reticence. Findings demonstrated that “all the teacher participants held a negative view of the reticent students. They attributed reticence to more student internal causes within the student's control and willingness to communicate to more external, teacher controllable causes” (p. 119). I am impressed by how the author meticulously takes into account such factors as credibility, member checking, trustworthiness, etc., but it could have been more robust if the author had reported the inter-coder agreement index. Chapter 7 discusses the predictive role of extroversion, as a personality trait, on the Polish students' readiness to communicate in L2. The chapter concludes that such factors as “self-perceived levels of foreign language skills and language anxiety” (p. 135) can have a bearing on WTC in L2.

Investigating the role of flipped classroom strategy (FCS) among Iranian EFL learners' WTC is the focus of Chapter 8. The results of this mixed-methods study reveal that FCS affected learners' WTC by “making language learning enjoyable, increasing motivation, and decreasing language anxiety” (p. 155). Drawing on the experience sampling method, Chapter 9 reports on the dynamic interplay between WTC, foreign language anxiety (FLA), and foreign language enjoyment (FLE) among Iranian EFL students. The chapter concludes that a significant amount of variability is observed over time and that “the correlations between WTC and enjoyment were remarkably consistent, strong, and positive” (p. 169). In a conceptually-driven contribution, Chapter 10 foregrounds the pivotal role of a social network approach in L2 WTC. The authors elaborate on the key principles of the social network theory (SNT) and argue how these tenets can be viably employed to investigate WTC in L2.

Chapter 11 represents how Iranian EFL teacher immediacy, self-disclosure, and technology policy can predict students' L2 WTC. The chapter concludes that reducing the distance between the teacher and the students can play an important role in students' WTC in the classroom. If the authors had added more qualitative data, the findings could have benefited the readers more. The penultimate chapter elucidates how vocabulary size can have a bearing on L2 WTC in a Turkish EFL context. The author concludes that “vocabulary level of the participants significantly predicted their WTC inside the classroom” (p. 250). The closing chapter provides some directions for future research. The author calls for more studies to be undertaken on “different language skills, linguistic, pedagogical, psychological, and technological issues” (p. 261) that can dynamically interact with or impact students' WTC.

As an applied linguist, I believe that this compendium offers a lot of food for thought for language teachers, pre-service and in-service teachers, teacher educators, and syllabus designers who are interested to scrutinize the multifaceted nature of WTC in L2. However, had the editors included studies from other Asian countries, it would have been more insightful. It is hoped that more theoretically-driven and pedagogically-oriented studies are undertaken to shed more light on how students' WTC can show its hidden dimensions.

## Author Contributions

The author confirms being the sole contributor of this work and has approved it for publication.

## Conflict of Interest

The author declares that the research was conducted in the absence of any commercial or financial relationships that could be construed as a potential conflict of interest.
